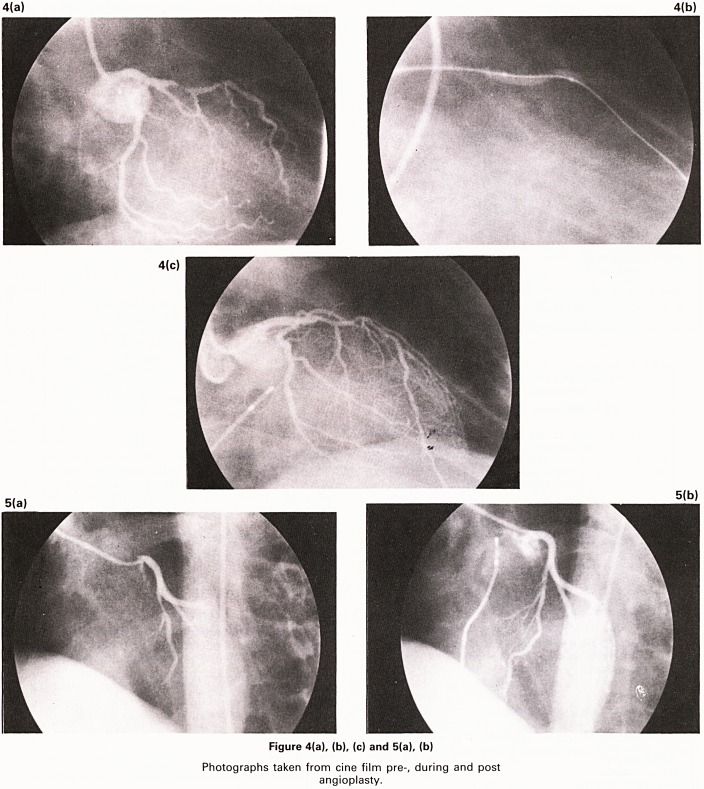# Percutaneous Coronary Angioplasty, the Bristol Experience

**Published:** 1986-10

**Authors:** Sylvia Aldridge, Mark Papouchado, Paul Walker, John Vann-Jones, Peter Wilde

**Affiliations:** Senior Radiological Registrar, BRI; Research Registrar, Cardiology Department, BRI; Consultant Cardiologist, Southmead Hospital; Consultant Cardiologist, BRI; Consultant Radiologist, BRI


### Percutaneous transluminal coronary angioplasty — the Bristol experience of the first 32 cases, June 1984 to December 1985

**Sylvia Aldridge, BSc (Hons), MB, ChB, MRCP, FRCR**
Senior Radiological Registrar, BRI.
**Mark Papouchado, MA, MB, BChir, DA, MRCP**
Research Registrar, Cardiology Department, BRI.
**Paul Walker, MB, BS, MRCP**
Consultant Cardiologist, Southmead Hospital.
**John Vann-Jones, PhD, MRCP**
Consultant Cardiologist, BRI.
**Peter Wilde, MB, ChB, MRCP, FRCR**
Consultant Radiologist, BRI.

### INTRODUCTION

The concept of using an arterial catheter as a therapeutic tool was introduced by Dotter and Judkins in 1964. They described a technique of using dilating catheters to increase the calibre of arteriosclerotic vessels in the lower limbs of patients who presented with signs and symptoms of severe ischaemia. Their initial results on 11 extremities in 9 patients showed that 6 patients' symptoms and signs improved while 3 patients remained unchanged. It was noteworthy that no patient showed any clinical deterioration. In their original paper they considered that severe proximal narrowing of the coronary arteries would be amenable to a manually guided dilator.

The application of this technique to stenotic coronary arteries proceed slowly for a number of reasons. There was concern about peripheral embolisation of atheromatous material, the possibility of immediate coronary occlusion resulting in a life-threatening situation and such technical problems as passing small dilating catheters through the coronary vessels.

The first percutaneous transluminal coronary angioplasty (PTCA) was performed by Gruntzig in Zurich in 1977. In 1979, Gruntzig et al described their initial experience with PTCA over an 18 month period. They had performed 53 PTCA in 50 patients: 46/53 were on native coronary arteries and 7/53 were on vein graft stenoses. They were successful in 32/50 patients (34/53 vessels) with a reduction in gradient from 58 to 19 mmHg and a reduction in stenosis from 84 to 34% area. They considered that the technique was limited by such anatomical factors as tortuosity of the artery, tightness and eccentricity of the stenosis and estimated that only 10–15% of candidates for coronary artery bypass grafting would be suitable for the alternative procedure of PTCA.

Since then over 10,000 patients have been treated worldwide by this technique.

This paper is a description of the initial experience in Bristol in the use of PTCA during an 18 month period June 1984 to December 1985 when 32 cases had been performed. Comparison of the Bristol results with those from elsewhere, the criteria for selection of patients for PTCA, the complications and the mechanism of PTCA are also discussed.

### PATIENT SELECTION AND TECHNIQUE

All patients had had previous coronary angiography for the evaluation of known or suspected coronary artery disease. The criteria for selection were:
1. Patients had to be fit for CABG surgery and have symptoms of ischaemic heart disease of sufficient severity despite full medical therapy to warrant intervention.
2. Patients had to have suitable angiographic anatomy of the lesion for PTCA (preferably single, proximal, short concentric lesion not involving large branches or the left main coronary artery).

### TECHNIQUE

The PTCA was performed as soon as possible after the initial coronary study but was usually after a 6–8 week delay. In some cases it was noted that there had been progression of the stenosis during this time.

The major technical problems in the technique of PTCA are:
1. Knowing exactly 'where you are' during the PTCA. This is achieved by the use of screening, previous angiograms and video recordings. Biplane equipment and screening is preferable but is not available at the present time at the BRI.
2. Having placed the Guiding catheter with its tip in the ostium of the coronary artery for PTCA (and having performed a pre-PTCA angiogram), the steerable guide wire is passed across the lesion to be dilated. At times due to the variable anatomy this cannot be achieved with ease or at all.
3. The deflated balloon catheter is then passed across the lesion. Again difficulties may be encountered at this stage of the technique.
4. The balloon catheter is then inflated for varying times and pressures according to circumstances and experience.
5. Pressure recordings are taken before, during and after the procedure if possible.
6. Finally a post PTCA angiogram is performed in several different projections to assess improvement in calibre and to detect any complications such as intimal dissection.

A temporary pacing wire is placed in the right ventricular outflow tract at the start of the procedure in the event of any rhythm disturbance occurring during the PTCA which might need immediate treatment. The tip of the wire further acts as an anatomical guide during the procedure in its relationship to the lesion to be dilated on the various projections used.

Drug treatment includes aspirin and dipyridamole started 24 hours before, sub-lingual nitrates, nifedipine and intracoronary nitrates during and heparin continued 12 hours after the PTCA.

The arterial sheath through which the catheters were introduced is left in place for 24 hours in case of the need for further investigation or treatment.

As an objective method of assessing the success of the procedure each patient had a pre and post PTCA exercise test using the modified Bruce protocol (treadmill with varying speeds and gradients). The pre test was performed a few days before the procedure and the post test about 6 weeks after the PTCA.

The 'Team' consists of Radiologists, Cardiologists, Radiographers, Cardiac technicians, Nursing staff and the Cardiac surgical unit in the event of requiring emergency CABG.

### RESULTS

In the 32 patients there were 34 attempted dilatations. (Table 1.) Of these, 21 were successful. With the exception of a vein graft dilatation all the successful procedures were on the left coronary artery anterior descending branch (19) or the diagonal branch of the LAD (1). This is a success rate of 77% on the left coronary artery angioplasties. None of the procedures on the circumflex or right coronary arteries were successful.

In the study there were 25 men and 7 women. Success was achieved in 18 of the men (Table 2) and 3 of the women (Table 3). The age distribution of all patients is shown in Fig 2.

The reasons for the 13 failed procedures are shown in Table 4 and include anatomical and technical problems.

The complications included 4 myocardial infarctions (12.5% of the 32 patients). Of these 2 required surgery, one as an immediate CABG and one after 10 days because of persisting symptoms and electrographic changes. (Table 5.)

The results of 7 of the exercise test performed pre- and post PTCA, where the test was positive initially and negative subsequently are shown in Table 6. The % reduction in luminal area is shown together with the reduction in pressure across the lesion.

In Fig. 3 the % change in calibre is correlated with the exercise test pre and post PTCA. The treadmill exercise times in minutes correspond to the time of occurrence of either chest pain or electrocardiographic changes necessitating cessation of exercise. If no pain or ecg changes has occurred at 15 minutes when the protocol is complete then the test is considered negative. Some patients

**Figure 1**

Schematic representation of the technique of Percutaneous transluminal coronary angioplasty.

**Figure 2**

Age distribution of 32 patients who had PTCA during an 18 month period, June 1984 to December 1985 in Bristol.

**Table 1**

**Bristol results of PTCA–vessels dilated.**

***Bristol results: 34 dilatations attempted in 32 patients***

| | Number | Successful |
|---|---|---|
| *LAD* | 25 | 19 |
| *Diagonal* | 1 | 1 |
| *Circumflex* | 3 | 0 |
| *RCA* | 3 | 0 |
| *CABG grafts* | 2 | 1 |

**Table 2**

**Results of PTCA in 25 men**

***25 Men***

***24 with single vessel attempted dilatation***
***1 with 2 vessel dilatation, circumflex and RCA***

Success in 18/25 men
LAD, 17/20 successful
RCA, 0/1
Circumflex, 0/1
Vein grafts, 1/2
2 vessel study – unsuccessful

**Table 3**

**Results of PTCA in 7 women**

***7 Women***

***6 with single vessel attempted dilatation***
***1 with 2 vessel dilatation, diagonal branch of LCA and circumflex***

Success in 3/7 women
LAD, 2/5 successful
RCA, 0/1
2 vessel study Diagonal successfully dilated, circumflex unsuccessful

**Table 4**

**Reasons for failed PTCA**

***Of 34 dilatations, 13 failures***

*LAD – 6 failures*

In 3 – guide wire would not cross lesion
In 2 – balloon would not cross lesion
In 1 – the balloon was inflated but position was lost

*Circumflex – 3 failures*

In 1 – balloon would not cross lesion
In 1 – occlusion at 45 minutes after successful dilatation
In 1 – poor angiographic results. MI after 48 hours

*RCA – 3 failures*

In 1 – guide wire would not cross lesion
In 1 – dissection of RCA with CABG at 10 days
In 1 – attempted dilatation resulted in hypotension and occlusion

*Vein graft – 1 failure*

In 1 – balloon would not cross lesion

**Table 5**

**Complications of PTCA**

***Of 34 dilatations in the 32 patients***

| | |
|---|---|
| *Successful* | Single vessel dilatation *20/30* |
| | Two vessel dilatation in 2 patients *1/4* |
| *CABG* | *2,* 1 as emergency, 1 after 10 days |
| *MI* | *4,* 1 needing emergency CABG |
| | 1 with CABG at 10 days |
| | 1 at 48 hours |
| | 1 with subendocardial lateral MI |
| *Deaths* | *None* |

**Table 6**

**Exercise testing–% reduction in lumen and pressure reduction**

***14 Exercise tests performed Pre and Post PTCA***
***7 Positive Pre and Negative Post PTCA***

| *Patient* | *% reduction in lumen* | *Pressure reduction* |
|---|---|---|
| B.P. Preseptal | 99 –50 | 60– 5 |
| P.G. Preseptal | 90 – 0 | 60– 5 |
| L.C. 1st S involved | 95L–50 | 45–40 |
| D.P. After 1st S | 99 –60 | 25–15 |
| A.B. Preseptal | 95 – 0 | 40– 0 |
| D.M. Preseptal | 99 –60 | 50– 0 |
| R.G. 1st D involved | 95 –50 | |

had to stop before that time, however, because of other symptoms, e.g. breathlessness. It can be seen that in nearly all cases there has been an increase in length of time on the treadmill comparing pre and post PTCA.

Two examples of successful Percutaneous transluminal coronary angioplasties are shown in Fig 4 and Fig 5.

In Fig 4a, the pre-angioplasty angiogram shows a tight stenosis on the left anterior descending coronary artery (a right anterior oblique view of the heart). In Fig 4b, the inflated balloon is in position at the site of the stenosis. In Fig 4c, there has been almost complete dilatation of the stenosis.

In Fig 5a and b, a stenosis is again seen on the proximal left anterior descending coronary artery (this time in the left anterior oblique view). On the post angioplasty film there has been considerable improvement in the calibre of the vessel at the site of the previous stenosis. A linear translucency corresponding to an intimal flap can be seen at the site of dilatation. In the absence of symptoms or signs this is not of any serious significance being seen frequently post PTCA.

**Figure 3**

Percentage change in luminal calibre against change in time on Treadmill exercise testing pre- and post PTCA.

### DISCUSSION

Comparison with other centres performing PTCA (Table 7) shows that Bristol has similar results both in the success rate and the number of patients requiring CABG. It is noteworthy that there were no deaths in the Bristol series, although our numbers are smaller than some other series. The % complication of myocardial infarction is higher than other studies. This includes a case where the infarct was considered to be subendocardial and small and where there was a good angiographic and symptomatic result post PTCA. The results show that the left anterior descending coronary arteries angioplasties have a considerably higher success rate than either the right or circumflex coronary arter angioplasties. This is comparable with other centres.

4(a) 4(b) 4(c) 5(a) 5(b)

**Figure 4(a), (b), (c) and 5(a), (b)**

Photographs taken from cine film pre-, during and post angioplasty.

The ideal lesion for successful PTCA has the following characteristics:

1. Proximal site of stenosis – for ease of manipulation of the catheter through the lesion.
2. Concentric stenosis – increased risk of complications with tapered lesions.
3. Discrete stenosis.
4. Non-calcified lesion.
5. No involvement of large side branches (more than 2 mm in diameter) since occlusion of such vessels is likely to result in myocardial infarction.
6. The stenosis should be more than 50% and less than 95% to allow passage of the catheter through the lesion.
7. Absence of coronary spasm on the pre PTCA angiogram.
8. Lesion of the left anterior descending coronary artery since angioplasty at this site is associated with a decreased mortality compared with the right or circumflex coronary angioplastics.

The complications of PTCA may be divided into two groups: those due to the technique of arteriography and those due specifically due to coronary angioplasty.

In the first group are included such complications such as systemic embolisation, arterial occlusion, neurological disturbances, local haemorrhage at the catheter site and contrast media reactions.

**Table 7**

**Bristol results compared with other series**

| | Number of patients | Number of vessels | Success in patients (%) | Success in vessels | CABG | MI | Deaths | Recurrence |
|---|---|---|---|---|---|---|---|---|
| Gruntzig *et al.*, 1979, Zurich | 50 | 53 | 32 (64%) | 34 | 5 (10%) | 3 (6%) | 1 (2%) | 3/27 studied (11%) |
| Multi-centre (34) in USA and Europe | 631 | | (59%) | | 40 (6%) | 29 (4%) | 6 (1%) | |
| Cowley *et al.*, 1981, USA | 25 | 29 | 14 (56%) | 18 (62%) | None | None | None | 3/14 studied (21%) |
| Cumberland *et al.*, 1985 3 centres, L, S, B | 240 | | 182 (76%) | | 16 (6.6%) | 20 (8%) | 3 (1.3%) | |
| Wilde *et al.*, Bristol, 1985 | 32 | 34 | 20 (62.5%) | 21 (62%) | 2 (6.25%) | 4 (12.5%) | None | |

a. Acute occlusion of the coronary artery due to either dissection, spasm or thrombosis. Cumberland et al in 1985 in their study of 240 PTCA had 20 (8%) cases with acute occlusion. They successfully treated occlusion due to spasm by immediate repeat angioplasty. With dissection, however, they considered that repeat angioplasty only worsens the situation often by canalisation of a false passage. In their study acute occlusion accounted for 14 of 16 CABG and 2 of the 3 deaths. They noted that females were at a higher risk of coronary occlusion than males (23% compared with 6.4%). It has been noted that PTCA of the right coronary artery or on 2 more vessels is associated with an increased risk or occlusion.

b. Persisting coronary insufficiency due to failure of successful dilatation of the lesion. This may result in persisting angina, or miocardial infarction and require CABG at a future date.

c. Delayed spasm or rhythm disturbances associated with the dilated vessel. This has been noted particularly in those patients who had had angioplasty of the left main coronary artery.

d. Recurrence of the stenosis. This is reported in 14–47% of cases. There is increased risk of Restenosis within 4–6 months of the procedure, in males, in angioplasty of bypass graft stenoses, in patients with severe angina before the procedure. Repeat coronary angioplasty has been successfully performed in 85% of cases of restenosis.

The mortality of PTCA is 0.9%. This is higher than the figure quoted for CAGB, of 0.4%. The adverse factors are: female gender (tend to be older with more severe and unstable angina), previous CABG, the presence of left main coronary disease, dilatation of the right or circumflex coronary arteries, an attempt at dilating a vein graft stenosis, longer duration of angina and age more than 60 years.

The subjective symptomatic improvement experienced by patients after PTCA suggests that there is improved coronary blood flow to ischaemic myocardium. However, the post PTCA angiogram is not always a good indicator of this and non-invasive tests such as Treadmill exercise tests and Thallium scintigraphy are used to demonstrate indirectly improved left ventricular myocardial perfusion. The Bristol results in 14 patients who were exercised pre and post PTCA according to the modified Bruce protocol showed that 7 were markedly improved after angioplasty.

The mechanism of angioplasty was studied by Block who showed in 1980 that there is Edothelial desquamation and shearing of superficial plaque elements in all areas where the angioplasty balloon is inflated. In areas of moderate vascular stenosis, re-endothelialisation and healing Fintima results in an enlarged lumen. In more tightly stenosed vessels, where the size of the narrowed arterial lumen and inflated angioplasty balloon are more disparate splitting of the plaque occurs. In some cases, the split extends to the internal elastic membrane. This focal controlled injury to the plaque causes an immediate increased luminal size. Due to such splits, angiography after PTCA may demonstrate an irregular edge of the column of contrast media at the site of dilatation and may even show filling defects. This should not be considered as a dissection unless there is evidence of the latter clinically. As fibrosis and healing occurs, retraction and endothelialisation of the separated intimal flaps further enlarges the lumen and smooths its inner contour in a period of 2 to 3 weeks.

### CONCLUSION

Thus it has been shown in this paper that although this initial Bristol series of PTCA's is small, the results are comparable to other centres.

It has been shown also that in selected cases it is an excellent alternative to CABG, both in the cost-saving aspect to the N.H.S. (considerably shorter bed-time and equipment usage) and benefit to the patient (obviating the need for major surgery and improving life style).

It is considered that the increasing experience of the team and the continuing careful selection of suitable cases will minimise the complications of the technique of Percutaneous transluminal coronary angioplasty.

### ACKNOWLEDGEMENT

We wish to thank the Medical Illustration department of the Bristol Royal Infirmary for their work in this paper.

### REFERENCES

DOTTER, C. T. and JUDKINS, M. D. (1964) Transluminal treatment of arteriosclerotic obstruction. Description of a new technique and a preliminary report of its application. *Circulation* **30**, 654–670.

GRUNTZIG, A. R. SENNING, A. and SIEGENTHALER, W. E. (1979) Non-operative dilatation of coronary artery stenosis. Percutaneous transluminal coronary angioplasty. *The New England Journal of Medicine* **301**, 61–67.

SHIU, M. F. SILVERTON, N. P. OAKLEY, D. and CUMBERLAND, D. (1985) Acute coronary occlusion during percutaneous transluminal coronary angioplasy. *British Heart Journal* **54**, 129–133.

BLOCK P. C. (1980) Percutaneous Translumional Coronary angioplasty. *A.J.R.* **135**, 955–957.

The American Journal of Cardiology. Proceedings of the National Heart, Lung and Blood Institute workshop on the outcome of PTCA. June 7–8. 1983. June 1984.

## Figures and Tables

**Figure 1 f1:**
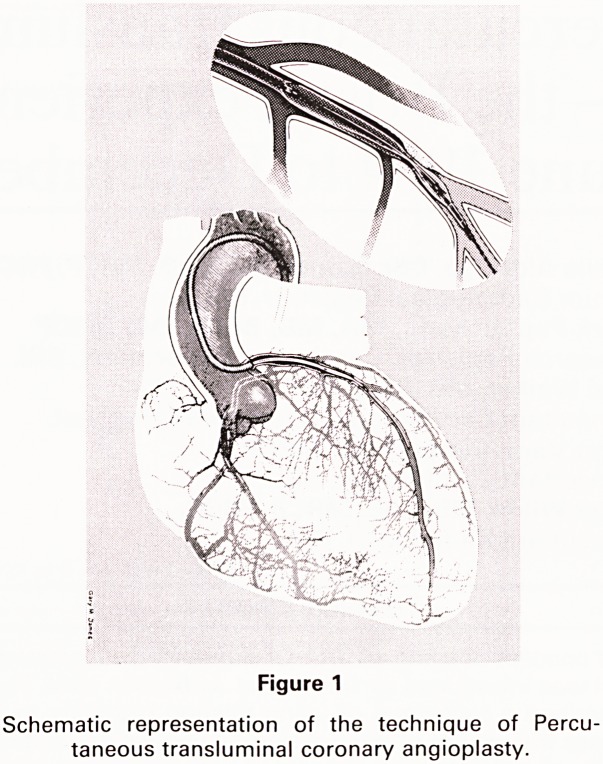


**Figure 2 f2:**
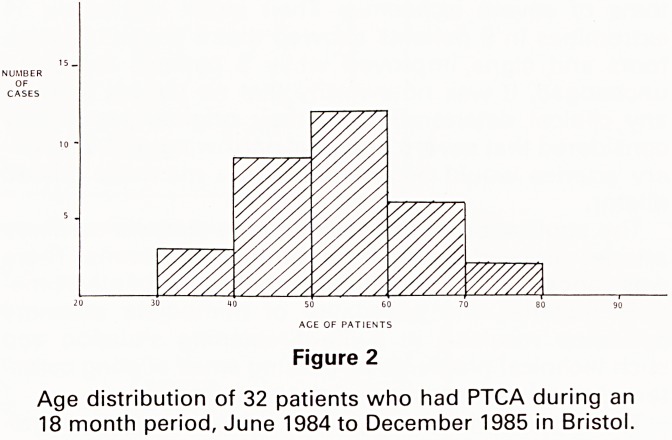


**Figure 3 f3:**
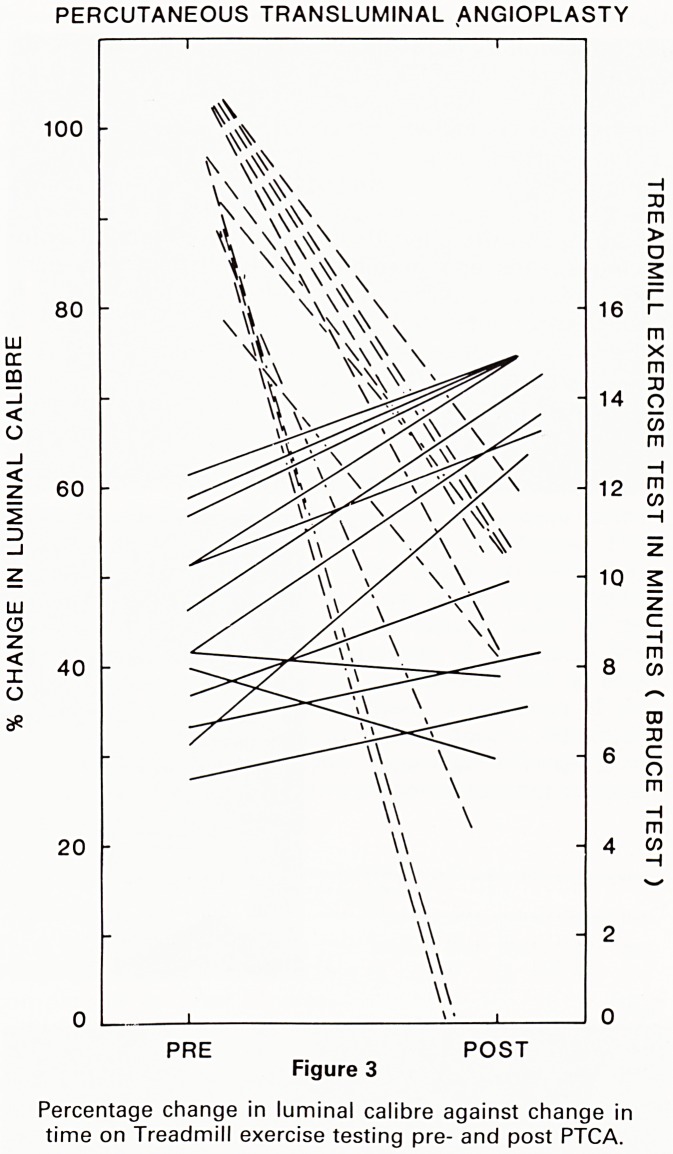


**Figure 4(a), (b), (c) and 5(a), (b) f4:**